# Thai-Lepto-on-admission probability (THAI-LEPTO) score as an early tool for initial diagnosis of leptospirosis: Result from Thai-Lepto AKI study group

**DOI:** 10.1371/journal.pntd.0006319

**Published:** 2018-03-19

**Authors:** Theerapon Sukmark, Nuttha Lumlertgul, Sadudee Peerapornratana, Kamol Khositrangsikun, Kriang Tungsanga, Visith Sitprija, Nattachai Srisawat, Thai-Lepto AKI study group

**Affiliations:** 1 Thungsong hospital, Nakhon Si Thammarat, Thailand; 2 Division of Nephrology, Department of Medicine, Faculty of Medicine, Chulalongkorn University, and King Chulalongkorn Memorial Hospital, Bangkok, Thailand; 3 Center for Critical Care Nephrology, The CRISMA Center, Department of Critical Care Medicine, University of Pittsburgh School of Medicine, Pittsburgh, Pennsylvania, United States of America; 4 Maharaj Nakhon Si Thammarat hospital, Nakhon Si Thammarat, Thailand; 5 Queen Saovabha Memorial Institute, Thai Red Cross, Bangkok, Thailand; Centers for Disease Control and Prevention, UNITED STATES

## Abstract

**Background:**

Leptospirosis is one of the most important zoonosis in the tropics. Currently, specific laboratory diagnostic test for leptospirosis such as polymerase chain reaction (PCR) or direct culture cannot be applied at the primary care setting especially in the resource- limited countries. Therefore, clinical presentation and laboratory examination are still the primary diagnostic tools for leptospirosis.

**Objectives:**

To detect clinical factors for predicting leptospirosis in suspected cases, and to create a clinical prediction score (THAI-LEPTO) that is practical and easy to use in general practice while awaiting laboratory results.

**Materials and methods:**

We performed a prospective multicenter study with a development and a validation cohort of patients presenting with clinical suspicion of leptospirosis as per the WHO clinical criteria. The development cohort was conducted at 11 centers in 8 provinces around Thailand. The validation cohort was conducted at 4 centers in 1 province from the Northeastern part of Thailand. Leptospirosis confirmed cases were defined if any one of the tests were positive: microscopic agglutination test, direct culture, or PCR technique. Multivariable logistic regression was used to identify predictors of leptospirosis. The clinical prediction score was derived from the regression coefficients (original) or from the odds ratio values (simplified). We used receiver operating characteristic (ROC) curve analysis to evaluate the diagnostic ability of our score and to find the optimal cutoff values of the score. We used a validation cohort to evaluate the accuracy of our methods.

**Results:**

In the development cohort, we enrolled 221 leptospirosis suspected cases and analyzed 211. Among those, 105 (50%) were leptospirosis confirmed cases. In logistic regression adjusted for age, gender, day of fever, and one clinical factor at a time, leptospirosis group had more hypotension OR = 2.76 (95% CI 1.07–7.10), jaundice OR = 3.40 (95%CI 1.48–8.44), muscle pain OR = 2.12 (95%CI 1.06–4.26), acute kidney injury (AKI) OR = 2.90 (95%CI 1.31–6.15), low hemoglobin OR = 3.48 (95%CI 1.72–7.04), and hypokalemia with hyponatremia OR = 3.56 (95%CI 1.17–10.84) than non-leptospirosis group. The abovementioned factors along with neutrophilia and pulmonary opacity were used in the development of the score. The simplified score with 7 variables was the summation of the odds ratio values as follows: hypotension 3, jaundice 2, muscle pain 2, AKI 1.5, low hemoglobin 3, hypokalemia with hyponatremia 3, and neutrophilia 1. The score showed the highest discriminatory power with area under the curve (AUC) 0.82 (95%CI 0.67–0.97) on fever day 3–4. In the validation cohort we enrolled 96 leptospirosis suspected cases and analyzed 92. Of those, 69 (75%) were leptospirosis confirmed cases. The performance of the simplified score with 7 variables at a cutoff of 4 was AUC 0.78 (95%CI 0.68–0.89); sensitivity 73.5; specificity 73.7; positive predictive value 87.8; negative predictive value 58.3.

**Conclusions:**

THAI-LEPTO score is a newly developed diagnostic tool for early presumptive diagnosis of leptospirosis in patients presenting with severe clinical suspicion of the disease. The score can easily be applied at the point of care while awaiting confirmatory laboratory results. Each predictor used has been supported by evidence of clinical and pathophysiological correlation.

## Author summary

Leptospirosis is an important zoonosis in the tropics. However, there is still a major barrier of early diagnosis of leptospirosis due to a lack of specific and sensitive testing. Moreover, in resource-limited countries with small budgets, a simple and cheap diagnostic test is essential for the diagnosis of leptospirosis. The THAI-LEPTO score has been developed and validated based on the relevant clinical history and related laboratory test. This score will help the physician in early diagnosis and prompt treatment leptospirosis as a point of care test. Finally, we aim that this scoring system can be one of the key parts of diagnostic algorithm and improve leptospirosis patients’ outcome.

## Introduction

Leptospirosis is a common zoonosis that can occur worldwide but is mostly found in tropical regions. Several mammals are natural hosts and humans are infected after environmental or animal contact. In Thailand, after a severe flood in 2000 a large outbreak of leptospirosis occurred and resulted in a total of 14,285 cases or 23 cases per 100,000 population. The background prevalence of leptospirosis in Thailand has been 3,000–4,000 cases per year (or 5 cases per 100,000 population) [1].

Inexperienced physicians are reluctant to make a presumptive diagnosis of leptospirosis because its clinical presentation can be similar to many other infectious diseases such as rickettsial infection, dengue hemorrhagic fever, malaria, influenza, and bacterial sepsis. To alleviate disease severity early diagnosis is key in allowing early treatment. However, current diagnostic tests such as direct culture, rapid tests for antibody detection, and other serological tests are insensitive during the early phase of leptospirosis infection[2–5]. Moreover, polymerase chain reaction (PCR) testing is still expensive and difficult to apply in a primary health care setting. Inevitably, at the point of care, most physicians still need to use initial clinical presentations as the primary tool to diagnose leptospirosis. Unfortunately, the diagnostic performance of Faine’s criteria [6] for presumptive diagnosis of leptospirosis, endorsed by the World Health Organization (WHO) since 1982, or other following modified versions are variable [7–10].

The present study aimed to detect clinical factors for predicting the diagnosis of leptospirosis in suspected cases, and to create a clinical prediction score that is practical and easy to use in general practice while awaiting confirmatory laboratory results.

## Methods

### Ethics statement

The study protocol was approved by the Institutional Review Board of Faculty of Medicine, Chulalongkorn University (IRB number 137/55), and the Institutional Review Board of Ministry of Public Health of Thailand (IRB number 005/2017). Participants consented for participation in the protocol.

### Patients and study design

We performed a prospective multicenter study with a development and a validation cohort of patients presenting with clinical suspicion of leptospirosis as per the WHO clinical criteria [11]. Specific inclusion criteria included high fever (body temperature higher than 38°C), severe myalgia, and history of exposure to reservoir animals or flood water. Exclusion criteria included patients who suffered from other known infectious diseases. The development cohort was conducted at 11 centers in 8 provinces around Thailand during 2012 to 2014. Blood and urine were serially collected on the first three days after enrollment and on day 7. The validation cohort was conducted at 4 centers from the Northeastern part of Thailand during 2015 to 2016. Blood and urine were collected on the first day after enrollment and on day 7. We applied the same inclusion and exclusion criteria to both the development and validation cohorts.

### Sample collection

Twelve milliliters of blood and a 30 mL urine sample were taken from leptospirosis suspected patients on the first day of enrollment. Urine samples were poured into 50 ml conical centrifuge tubes. Both plasma and urine were centrifuged for 10 minutes at 1000g at 4°C, and frozen at -20°C until shipped to the central laboratory. Samples were then stored at -80°C until analyzed.

### Definitions

We used three standard techniques to confirm leptospirosis: microscopic agglutination test (MAT), direct culture, and PCR technique. Briefly, MAT was performed using the standard protocol from the WHO guideline [11]. A positive MAT was defined as a single serum titer of ≥ 1:400 or a 4-fold rise in pair serum. For direct culture of leptospires, one drop of whole blood was cultured in 4 mL liquid Elling hausen–McCullough–Johnson–Harris at 29 °C for 2 weeks. Detection of leptospires was accomplished by direct observation using dark-field microscopy. For PCR technique, DNA was extracted from urine samples using a High Pure PCR Template Preparation kit (Roche Diagnostics, Germany). The two primers used for amplification of LipL 32 gene were as follows: 45F primers (5’ AAG CAT TAC CGC TTG TGG TG3’) and 287R primers (5’ CGA ACT CCC ATT TCAGCG AT 3’). PCR reactions of urine samples were performed in a final volume of 20 μL, corresponding to 2 μL of genomic DNA and 18 μL of reaction mix containing 25 mM of each dNTP, 0.1 μL of Taq DNA polymerase, 0.4 μL of each primer in 25 mM MgCl_2_ and 10x KCl under 13.5 μL DW. The PCR program consisted of an initial cycle of 94°C for 10 min; followed by 40 cycles of each 94°C for 1 min, 55°C for 1 min, and 72 °C for 1 min; and a final extension step at 72°C for 7 min. PCR products were run on 1% agarose gel with ethidium bromide and photographed. We used the term *“Leptospirosis suspected cases”* for patients who were enrolled into the study, and *“Leptospirosis confirmed cases (Leptospirosis)”* if any one of the above tests was positive.

### Statistical analyses

Statistical analyses were performed using SPSS software, version 17.0, with statistical significance set at *p*<0.05. Comparisons between leptospirosis and non-leptospirosis groups were performed with Pearson’s Chi-Square asymptotic test or Fisher’s Exact test for categorical variables and using Student’s *t*-Test or Kruskal-Wallis one-way analysis of variance by ranks for continuous variables.

In a development cohort, we considered 14 clinical factors and conducted univariate and multivariable logistic regression to identify significant predictors of leptospirosis. In the multivariable models we looked at each clinical factor at a time and adjusted for age, gender, and fever day. Next, based on the adjusted models, we identified 6 clinical factors that were significant predictors of leptospirosis and, because of clinical importance, considered 2 additional factors that did not have statistical significance to use in developing a clinical score for leptospirosis. The likelihood ratio test, Hosmer-Lemeshow goodness of fit test, and Nagelkerke pseudo R-Square were used in selecting the two multivariable models that were the basis for developing our clinical score. Specifically, we used a model based on all 8 clinical factors (model 1), and a model based on the 6 significant factors plus one nonsignificant but clinically important factor (model 2). For each of the 2 models we created two prediction scores as follows: 1) a score based on the exponential equation 1/(1+*e*^*-z*^), where Z is the sum of all parameter estimates from the multivariable logistic regression given that a condition was present (original THAI-LEPTO); 2) a simplified score by adding the adjusted odds ratio values, rounded to the nearest 0.5, given that a condition was present (simplified THAI-LEPTO) [12]. We used receiver operating characteristic (ROC) curve analysis to evaluate the diagnostic ability of our scores. We compared the area under the curve (AUC), sensitivity, specificity, likelihood ratio, and post-test probability values to find the optimal cutoff values of the THAI-LEPTO scores. Furthermore, we evaluated our models at different days from fever onset.

Finally, we used a validation cohort to evaluate the accuracy of our methods. IgM rapid test performance statistics were used as a comparison group in both the development and validation cohorts.

## Results

**Fig 1:**
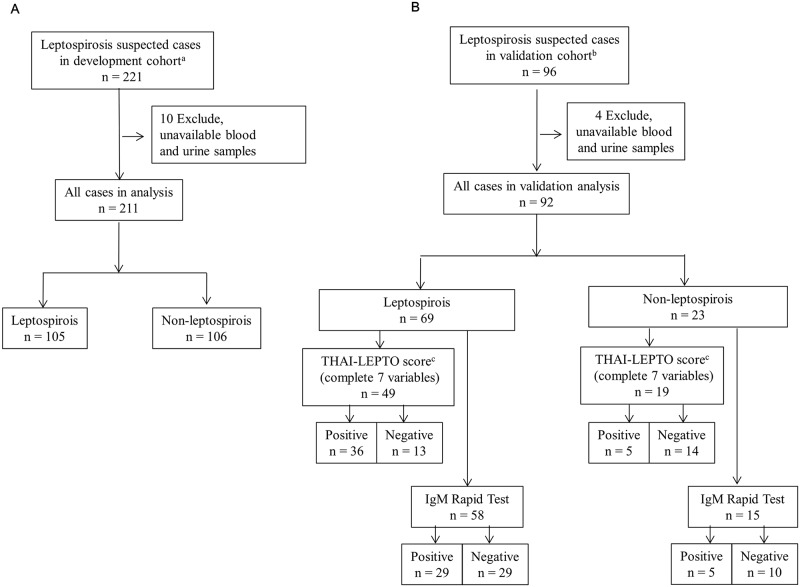
Flow chart of subject disposition for the THAI-LEPTO score study in development cohort (A) and validation cohort comparing between THAI-LEPTO score and IgM rapid test (B). ^a^Development cohort in 11 centers during 2012 to 2014. ^b^Validation cohort in 4 centers during 2015 to 2016.^c^Simplified THAI-LEPTO score model II (7 variables) with cutoff value of 4 was used in the validation analysis.

In the development cohort, we enrolled 221 leptospirosis suspected cases of which 211 were analyzed. Among those, 105 (50%) were leptospirosis confirmed cases and 106 (50%) were confirmed to not have leptospirosis (Fig 1A). In the validation cohort 96 leptospirosis suspected cases were enrolled but only 92 were analyzed. Of those, 69 (75%) were leptospirosis confirmed cases and 23 (25%) were confirmed to not have leptospirosis (Fig 1B). The three most common serovars in our pulled cohort were Shermani (55.3%), Australis (12.8%), and Sejroe (4.3%).

### Development cohort analysis

For the development cohort, comparisons of clinical, laboratory and radiographic characteristics between leptospirosis and non-leptospirosis groups at the time of enrollment are shown in Table 1. Leptospirosis patients had mean age of 42.8 years old, were predominantly male (81.9%), and worked as farmers (62.1%). There was no difference in body weight, occupation, comorbid conditions, and epidemiologic exposures (mostly flood exposure) between the leptospirosis and non-leptospirosis groups.

**Table 1:** Patients’ clinical and laboratory characteristics (on admission) of confirmed-leptospirosis and non-leptospirosis group in development cohort (n = 211).

Clinical characteristics	Leptospirosis (n = 105)	Non-Leptospirosis (n = 106)	P-value
Age, years, (SD)	42.8 (15.2)	43.2 (16.1)	0.86
Male gender, n (%)	86 (81.9)	80 (75.5)	0.25
Body weight, kg, (SD)	58.9 (12.5)	61.0 (10.2)	0.25
Height, cm., (SD)	164.6 (7.5)	165.3 (9.2)	0.62
Body Mass Index, kg/m^2^ (SD)	21.7 (4.1)	22.4 (3.2)	0.26
Occupation	(n = 100)	(n = 87)	0.21 ^c^
Farmer, n (%)	54 (62.1)	59 (59.0)	
Worker, n (%)	21 (24.1)	24 (24.0)	
Business, n (%)	1 (1.1)	4 (4.0)	
Public servants, n (%)	1 (1.1)	0	
Housewife/householder,n(%)	3 (3.4)	0	
Student, n (%)	3 (3.4)	10 (10.0)	
Unemployment, n (%)	3 (3.4)	2 (2.0)	
Monk, n (%)	1 (1.1)	1 (1.0)	
Currently tobacco use, n (%)	42/98 (42.9)	24/92 (26.1)	0.015*
Alcoholism, n (%)	6/103 (5.8)	3/102 (2.9)	0.50 ^c^
Co-morbidity disease			
Diabetes, n (%)	5/103 (4.9)	3/102 (2.9)	0.72 ^c^
Hypertension, n (%)	8/103 (7.8)	2/102 (2.0)	0.10 ^c^
Dyspidemia, n (%)	4/104 (3.8)	3/102 (2.9)	>0.99^c^
Malignancy, n (%)	1/103 (1.0)	0/102 (0.0)	>0.99^c^
Anemia, n (%)	2/102 (2.0)	1/101 (1.0)	>0.99^c^
Gout, n (%)	2/102 (2.0)	1/101 (1.0)	>0.99 ^c^
Asthma/COPD, n (%)	3/103 (2.9)	1/102 (1.0)	0.62 ^c^
CKD, n (%)	2/102 (2.0)	1/101 (1.0)	>0.99^c^
Chronic liver disease, n (%)	4/103 (3.9)	1/102 (1.0)	0.37
Exposure			
Flood, n (%)	69/104 (66.3)	65/99 (65.7)	0.92
Animal, n (%)	11/104 (10.6)	8/99 (8.1)	0.54
Symptoms and signs			
Days of fever, day (Q1,Q3)	4 (3, 5)	3 (2, 4.25)	0.034*
Body temperature, °C (SD)	38.1 (1.1)	38.4 (1.1)	0.039*
Jaundice, n (%)	34/104 (32.7)	14/102 (13.7)	0.001*
Nausea, n (%)	36/104 (34.6)	41/102 (40.2)	0.41
Vomiting, n (%)	31/104 (29.8)	25/102 (24.5)	0.39
Headache, n (%)	65/104 (62.5)	65/102 (63.7)	0.86
Photophobia, n (%)	6/104 (5.8)	3/102 (2.9)	0.50^c^
Malaise and fatigue, n (%)	78/104 (75.0)	77/102 (75.5)	0.94
Alteration of consciousness, n (%)	3/104 (2.9)	3/102 (2.9)	>0.99^c^
Skin lesion, n(%)	5/104 (4.8)	3/102 (2.9)	0.72 ^c^
Abdominal pain, n (%)	22/104 (21.2)	10/102 (9.8)	0.025*
Gross hematuria, n (%)	3/104 (2.9)	1/102 (1.0)	0.62 ^c^
Dysuria, n (%)	2/104 (1.9)	5/102 (4.9)	0.28 ^c^
Muscle pain, n (%)	37/104 (35.6)	20/102 (19.6)	0.01*
Convulsion, n (%)	1/104 (1.0)	1/102 (1.0)	>0.99^c^
Dyspnea, n (%)	14/104 (13.5)	7/102 (6.9)	0.12
Cough, n (%)	25/104 (24.0)	19/102 (18.6)	0.34
Hemoptysis, n (%)	4/104 (3.8)	3/102 (2.9)	0.99 ^c^
Hypotension, n (%)	21/104 (20.2)	7/102 (6.9)	0.005*
Diarrhea, n (%)	2 (1.9)	1 (0.9)	0.62 ^c^
Subconjunctival suffusion, n (%)	3 (2.9)	1 (0.9)	0.37 ^c^
Sore throat, n (%)	3 (2.9)	4 (3.8)	>0.99 ^c^
Systolic Blood Pressure, mmHg (Q1,Q3)	110(100, 121)	114 (100, 122)	0.12
Diastolic Blood Pressure, mmHg (Q1,Q3)	69.5 (60, 80)	70 (60, 80)	0.08
Laboratory values			
Hb, g/dL SD)	11.5± 2.4	13.2± 2.4	< 0.001*
WBC x10^3^, /μL (Q1,Q3)	9.1 (6.6, 13.7)	8.5 (5.5, 11.65)	0.18
PMN, % (Q1,Q3)	78.0 (70.0, 87.0)	74.65 (65.0, 84.8)	0.08
Platelet x10^3^ /μL (Q1,Q3)	132(53.25, 204.25)	156 (112, 246.5)	0.008*
Urine sp.gr (Q1,Q3)	1.015 (1.010, 1.025)	1.020 (1.015, 1.025)	0.046
Urine protein			0.23 ^c^
Negative n(%)	40 (38.1)	24 (22.6)	
Trace n(%)	9 (8.6)	11 (10.4)	
1+, n(%)	16 (15.2)	20 (18.9)	
2+, n(%)	11 (10.2)	12 (11.3)	
3+, n(%)	6 (5.7)	6 (5.7)	
> 3+, n(%)	1 (1.0)	0 (0.0)	
Urine RBC/HPF (Q1,Q3)	1 (0,2.25)	1 (0,2)	0.33
Urine WBC/HPF (Q1,Q3)	2 (1, 7.5)	1 (1, 3)	0.039*
Urine RBC cast (Q1,Q3)	0 (0, 0)	0 (0, 0)	0.25
Urine WBC cast (Q1,Q3)	0 (0, 0)	0 (0, 0)	0.40
Urine granular cast (Q1,Q3)	0 (0, 0)	0 (0, 0)	0.58
Total Bilirubin, μmol/L (Q1,Q3)	34.88 (14.11, 127.82)	23.43 (11.97, 63.27)	0.06
Direct Bilirubin, μmol/L (Q1,Q3)	23.00 (5.13, 84.65)	8.64 (5.10, 32.92)	0.11
Aspartate aminotransferase, μkat/L (Q1,Q3)	0.75 (0.43, 1.64)	0.95 (0.53, 2.61)	0.14
Alanine aminotransferase, μkat/L (Q1,Q3)	0.74 (0.48, 1.59)	1.01 (0.5, 1.91)	0.26
Serum Sodium, mmol/L (Q1,Q3)	135.0 (131.3, 138.0)	136.5 (131.8, 138.8)	0.54
Serum Potassium, mmol/L (Q1,Q3)	3.52 (3.29, 3.90)	3.70 (3.4F0, 4.06)	0.10
Serum bicarbonate, mmol/L (Q1,Q3)	21.4 (18.0, 25.3)	22.8 (18.0, 25.0)	0.66
Serum creatinine, μmol/L (Q1,Q3)	104.34 (77.81, 307.7)	83.11 (67.2, 107.87)	0.001*
AKI^a^, on admission, n (%)	37/104 (35.6)	17/104 (16.3)	0.002
AKI^a^, all cases^b^, n (%)	40 (38.1)	20 (18.9)	0.002
IgG rapid test^d^: positive, n (%)	2/50 (4.0)	5/48 (10.4)	0.26 ^c^
IgM rapid test^d^: positive, n (%)	25/64 (39.1)	20/62 (32.3)	0.43
Chest X-ray findings	(n = 53)	(n = 60)	0.18 ^c^
Normal, n (%)	40 (75.5)	52 (86.7)	0.13
Pulmonary opacities, n (%)	11 (20.8)	5 (8.3)	0.059
Pulmonary vascular congestion, n (%)	2 (3.8)	3 (5.0)	>0.99^c^
Electrocardiographic findings			0.08 ^c^
Normal, n (%)	30 (28.6)	46 (43.4)	0.025
Sinus tachycardia, n (%)	4 (3.8)	1 (0.9)	0.21 ^c^
ST-T abnormalities, n (%)	2 (1.9)	0 (0.0)	0.25 ^c^
Ectopic beats, n (%)	0 (0.0)	1 (0.9)	>0.99^c^
Tachyarrhythmia, n (%)	1 (1.0)	2 (1.9)	>0.99^c^
Bradyarrhythmia, n (%)	1 (1.0)	1 (0.9)	>0.99^c^

*Statistical significance

Abbreviations: AKI; acute kidney injury, CKD; chronic kidney disease, PMN; polymorphonuclear leucocyte, COPD; chronic obstructive pulmonary disease

^a^AKI, on admission, including AKI which presented on day of admission

^b^AKI, all cases, including AKI which presented on day of admission and during hospital stay

^c^ P-value from Fisher’s exact test

^d^Rapid test with lateral flow immunochromatographic assay, read by naked eye

**Fig 2:**
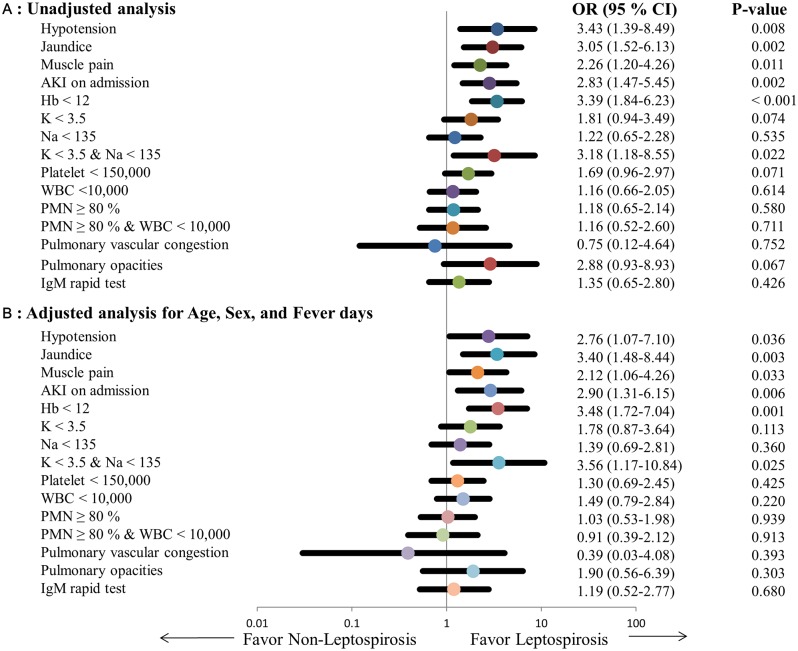
Forest plots of unadjusted (A) and adjusted (B) odds ratio in clinical prediction of leptospirosis, comparing with IgM rapid test in development cohort.

In logistic regression analysis adjusted for age, gender, day of fever, and one clinical factor at a time, we found that the leptospirosis group had more hypotension OR = 2.76 (95% CI 1.07–7.10), jaundice OR = 3.40 (95% CI 1.48–8.44), muscle pain OR = 2.12 (95% CI 1.06–4.26), acute kidney injury (AKI) OR = 2.90 (95% CI 1.31–6.15), low hemoglobin (Hb < 12 g/dL) OR = 3.48 (95% CI 1.72–7.04), and hypokalemia with hyponatremia (K < 3.5 mEq/L with Na < 135 mEq/L) OR = 3.56 (95% CI 1.17–10.84) than the non-leptospirosis group (Fig 2).

The abovementioned clinical factors along with neutrophilia and pulmonary opacity were used in the development of the THAI-LEPTO score. Model 1 contains all 8 variables. Model 2 omits pulmonary opacity containing only 7 variables. Table 2 contains the two models along with: 1) parameter estimates to be used in the original score; 2) rounded adjusted odds ratios to be used in the simplified score. Model 2 had a percentage of correct responses (that is, the overall percentage of correct positive and negative responses) of 73.0% with a Hosmer-Lemeshow p-value of 0.33 and a Nagelkerke R square of 0.28.

**Table 2:** Creating the THAI-LEPTO score of development cohort with multivariable regression-based method (n = 211).

Predictors	Model I (8 variables)			Model II (7 variables)		
	Parameter estimates^a^	Adjusted odds ratio^b^	Simplified score^c^	Parameter estimates^a^	Adjusted odds ratio^b^	Simplified Score^c^
Hypotension	1.301	3.674	3.5	1.030	2.802	3
Jaundice	0.302	1.352	1.5	0.813	2.256	2
Muscle Pain	0.791	2.205	2.0	0.614	1.847	2
AKI	0.387	1.473	1.5	0.416	1.516	1.5
Hb < 12 g/dL	1.063	2.896	3.0	1.028	2.796	3
K < 3.5 & Na < 135	1.440	4.221	4.0	1.126	3.082	3
PMNs ≥ 80% & WBC < 10,000/μL ^d^	0.617	1.853	2.0	0.097	1.102	1
Pulmonary opacity^d^	0.085	1.089	1.0	-	-	-
Constant	-1.852	0.157	-	-1.376	0.253	-

Abbreviations: AKI; acute kidney injury, Hb; hemoglobin, Na; sodium, K; potassium, PMN; polymorphonuclear leucocyte; WBC; white blood cell

^a^Parameter estimates (commonly known as unstandardized coefficients in multivariable regression model) for original THAI-LEPTO score equation.

Original THAI-LEPTO score (model I) equation is $\frac{1}{1+e^{-Z}}$, Z = -1.852 (in every case) +1.301 (if has “Clinical Hypotension”) + 0.302 (if has “Clinical jaundice) + 0.791 (if has “Muscle pain”) + 0.387 (if has “AKI”) + 1.063 (if has “Hb< 12”) + 1.440 (if has “K < 3.5 & Na < 135”) + 0.617 (if has “PMNs ≥ 80% & WBC < 10,000) + 0.085 (if has “Pulmonary opacity”)

(Z value was derived from summation of B values of regression coefficients multiply with each factor value (has the condition = 1, not has the condition = 0) in binary logistic regression models; -1.852 is the constant in equation; e value is 2.71828, as the mathematic constant.)

^b^Adjusted odds ratio for covariates in multivariable regression analysis

^c^Simplifiedscore closed to nearest integer of the adjusted odds ratio for simplified THAI-LEPTO score equation.

Simplifed THAI-LEPTO score is the sum of scores of the conditions; +3.5 (if has “Clinical Hypotension”) + 1.5 (if has “Clinical Jaundice”) + 2.0 (if has “Muscle pain”) + 1.5 (if has “AKI”) + 3 (if has “Hb < 12”) + 4 (if has K < 3.5 & Na < 135) + 2 (if has “PMNs ≥ 80% & WBC < 10,000”) + 1 (if has “Pulmonary opacity”), as show in S1 Fig

^d^Two predictors; pulmonary opacity and PMNs ≥ 80% & WBC < 10,000 were not obviously significant predictors in primarily uni-variable and multi-variable analysis (Fig 2), but these predictors could increase area under the ROC curve in analysis.

**Fig 3:**
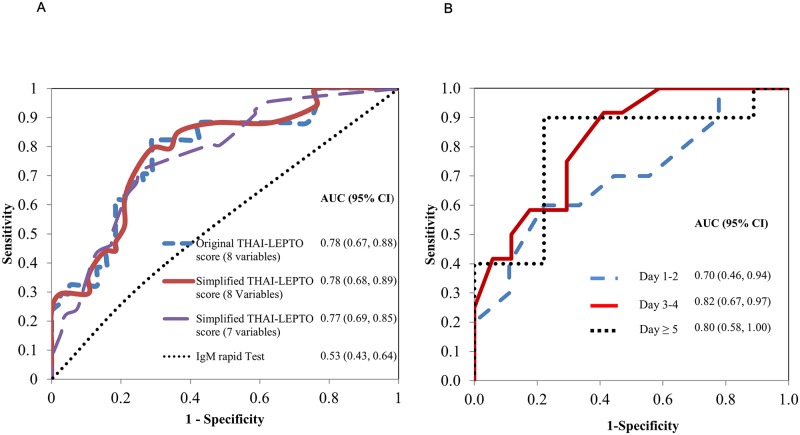
The area under the receiver operating characteristic (ROC) curve for predicting leptospirosis in each model of THAI-LEPTO scores, comparing with IgM rapid test (A) and according to fever days of simplified model (B) in development cohort study. AUC; area under ROC curve

The AUC were 0.78 (95%CI 0.67–0.88) for original THAI-LEPTO score model 1 at cutoff of 0.47, 0.78 (95%CI 0.68–0.89) for simplified score model 1 at cutoff of 5, and 0.77 (95%CI 0.69–0.85) simplified score model 2 at cutoff of 4 (Fig 3A). The AUC (95% CI) for model 2 stratified by days from fever onset were: 0.70 (0.46, 0.39) for < 3 days; 0.82 (0.67, 0.97) for 3–4 days; and 0.80 (0.58, 1.00) for > 4 days (Fig 3B).

Sensitivity, specificity, positive likelihood ratio and post-test probability at different cutoff values of the scores from the simplified models 1 and 2 compared with the IgM rapid test are shown in Table 3.

**Table 3:** THAI-LEPTO score of the development cohort at the best cutoff value to predict leptospirosis in suspected cases, comparing with IgM rapid test (n = 211).

Parameter/Models	Cutoff value	Sensitivity (%)	Specificity (%)	Positive Likelihood ratio	Positive, Post-Test Probability^a^
THAI-LEPTO score 8 variables (Simplified model I)	4.5	79.4	65.8	2.321	0.699
	5	79.4	71.1	2.743	0.733
	5.5	70.6	76.3	2.980	0.749
THAI-LEPTO score 7 variables (Simplified model II)	3.5	74.6	68.6	2.37	0.703
	4.0	71.6	74.3	2.786	0.736
	4.5	67.2	75.7	2.766	0.734
IgM rapid test^b^	0.519	39.1	67.7	1.21	0.548

^a^In condition with pre-test probability is 0.5

^b^Rapid test with lateral flow immunochromatographic assay, read by naked eye

### Validation cohort analysis

**Fig 4:**
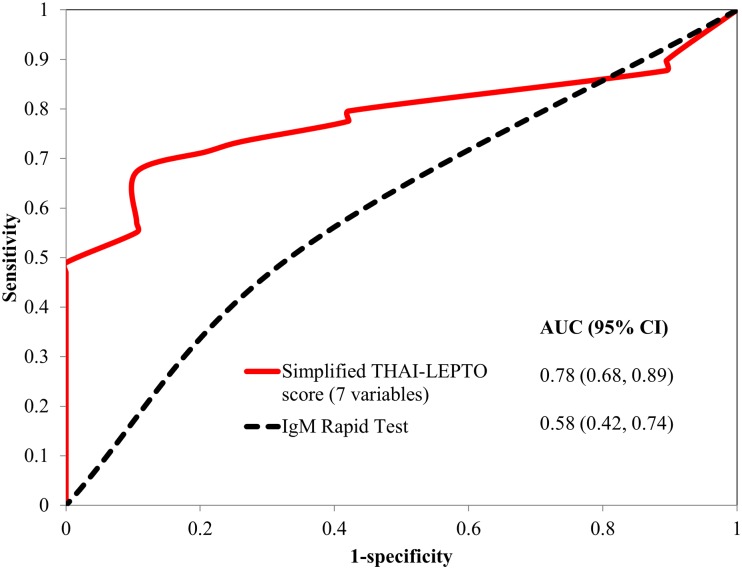
The area under the receiver operating characteristic (ROC) curve for predicting leptospirosis of simplified THAI-LEPTO score model II (7 variable), comparing with IgM rapid test in validation study. AUC; area under ROC curve.

For the validation cohort, the AUC of the simplified THAI-LEPTO score model 2 at cutoff of 4 and IgM rapid test were 0.78 (95%CI 0.68–0.89) and 0.58 (95%CI 0.42–0.74) respectively (Fig 4). The performance of the simplified score model 2 at a cutoff value of 4 versus that of the IgM rapid test was: 73.5 vs. 50.0 for sensitivity; 73.7 vs. 66.7 for specificity; 87.8 vs. 85.3 for positive predictive value; 58.3 versus 25.6 for negative predictive value; 2.79 vs. 1.5 for positive likelihood ratio; and 0.74 vs. 0.57 for positive post-test probability (Table 4).

**Table 4:** Accuracy of the simplified THAI-LEPTO score comparing with IgM rapid test in validation cohort analysis (n = 92).

Tools	Sensitivity (%)	Specificity (%)	PPV (%)	NPV (%)	Positive LR	Negative LR	Positive posttest probability^c^	Negative posttest probability^c^
THAI-LEPTO score^a^	73.5	73.7	87.8	58.3	2.79	0.36	0.74	0.26
IgM rapid test^b^	50	66.7	85.3	25.6	1.5	0.75	0.6	0.43

Abbreviations: PPV; positive predictive value, NPV; negative predictive value, LR; likelihood ratio

^a^Simplified THAI-LEPTO Score Model II (7 variables) at cutoff value = 4

^b^Rapid Diagnosis Test with lateral flow immunochromatographic assay, read by naked eye

^c^In condition with pre-test probability is 0.5

## Discussion

In summary, in patients presenting with clinical suspicion of leptospirosis we found that several clinical and laboratory parameters present on admission are associated with leptospirosis. Early hypotension, jaundice, muscle pain, AKI, low hemoglobin, and hypokalemia combined with hyponatremia were all associated with leptospirosis (Table 1). Additionally, neutrophilia and pulmonary opacity, although not significant predictors of leptospirosis in either univariate or multivariable analyses (Fig 2), when added to the multivariable models increased the AUC thus improving their predicting ability and providing a more accurate THAI-LEPTO score. Interestingly, THAI-LEPTO score had the highest discriminatory power (AUC 0.82, 95% CI 0.67–0.97) on days 3 or 4 after onset of fever (Fig 3B). Thus, THAI-LEPTO score is a useful tool in the early diagnosis of leptospirosis when rapid-serologic tests are not helpful.

Early diagnosis of leptospirosis is essential in improving outcomes. However, differentiating leptospirosis from other tropical infectious diseases such as dengue infection, or scrub typhus which share common clinical presentations is challenging to physicians. Moreover, current diagnostic tests including MAT, direct culture, and the IgM rapid test remain less sensitive and are of little help in decision-making during the early phase of infection [13]. Most of the time physicians use clinical manifestations and initial laboratory investigations to make a diagnosis and recommend treatment. THAI-LEPTO score should help physicians with limited access to diagnostic tools and varied clinical experience in diagnosing leptospirosis early. Even though some studies have well established clinical manifestations of leptospirosis[14–16] and another has recently proposed a diagnostic scoring model for leptospirosis[17]. To our knowledge, this is the first prospective study that develops and validates a score based on clinical and basic laboratory and radiographic characteristics present at the bedside to aid clinicians in the early diagnosis of leptospirosis.

In clinical practice the IgM rapid test is used for diagnosing leptospirosis. We thus compared the accuracy of the THAI-LEPTO score with that of the IgM rapid test applied to the validation cohort. Our score outperformed the IgM rapid test on all metrics used confirming its usefulness (Table 4). Clinical diagnostic scoring models have been developed and used in many countries because serologic tests are of less help in the early phase of infection. However, the accuracy of the previous models is variable. The performance of the simplified THAI-LEPTO score model 2 at a cutoff value of 4 is compared with other scoring models in Table 5. Although each model may have internal validity, the metrics reported in one study may not be generalizable outside the original population. Thus, comparisons between studies should be made while keeping study similarities in mind. With this in mind, between our scoring system and the recently reported scoring system from Sri Lanka [17], THAI-LEPTO score has higher specificity and positive likelihood ratio while Sri Lanka has better sensitivity and negative likelihood ratio.

**Table 5:** Performance of available diagnostic score models of leptospirosis.

Scoring model	THAI-LEPTO score	Rajakse S. et al’s model	Modified Faine’s Criteria with amendment (2012)	Modified Faine’s Criteria (2004)	Modified Faine’s Criteria (2004)	Faine’s Criteria (1982)
Validation study	2017^a^	2016[17]^b^	Jose LR. et al, 2016[8]^c^	Bandara K. et al, 2016[9]^d^	Bhatia M. et al, 2015[10]^e^	Chifou W. et al, 2010[7]
Country	Thailand	Sri Lanka	India	Sri Lanka	India	Thailand
Confirmatory tests	MAT Culture PCR	MAT	IgM ELISA	MAT PCR	MAT	MAT
AUC (95% CI)	0.78 (0.68, 0.89)	0.76	N/A	N/A	N/A	N/A
Sensitivity (%)	73.5	80.3	100	89.39	N/A	68
Specificity (%)	73.7	60.2	N/A	58.82	N/A	58
PPV (%)	87.8	54	N/A	58.42	21%	64
NPV (%)	58.3	84	N/A	89.55	N/A	59
Positive LR	2.79	2.01	N/A	2.17	N/A	N/A
Negative LR	0.36	0.32	N/A	0.18	N/A	N/A

Abbreviations: AUC; area under the ROC curve, MAT; microscopic agglutination test, PCR; polymerase chain reaction, ELISA; enzyme-linked immunosorbent assay, CI; confidential interval, PPV; positive predictive value, NPV; negative predictive value, LR; likelihood ratio, N/A; not available

^a^Simplified THAI-LEPTO score Model II (7 variables) at cutoff value = 4

^b^At cutoff value = 14

^c^The study mentioned the score only in115 cases of IgM ELISA positive, not in IgM ELISA negative cases.

^d^At the performance character of clinical history + epidemiologic history +rapid immunochromatographic test only

^e^Positive predictive values were 14.3, 6.5, and 8.7% for IgM rapid test, MAT, and IgM ELISA, respectively.

THAI-LEPTO score model 2 can be applied at the point of care as shown in S1 Fig. When a patient is clinically suspected of leptospirosis (WHO clinical description with epidemiologic exposure) they can be tested by the simplified score at the time of admission. If the patient’s score equals 4 or more, physicians can make a presumptive diagnosis of leptospirosis with a positive post-test probability of 0.74. However, other specific diseases such as hepatobiliary tract infection, bacterial sepsis, and malaria must be ruled out before making the initial diagnosis of leptospirosis.

THAI-LEPTO score is potentially easy to apply in clinical practice since it is derived from clinical manifestations and basic investigations. The parameters considered should be available in most hospitals even in resource-limited countries. However, THAI-LEPTO score may be less accurate (i.e. less sensitive and/or less specific) in detecting mild forms of leptospirosis which have non-specific clinical manifestations.

Interestingly, evidence of clinical and pathophysiological correlation between each predictor and leptospirosis has been documented. Hypotension, an early presenting sign of leptospirosis[18], may be the result of several factors, including initial systemic vasodilatation and increased vascular permeability secondary to cytokines and vasoactive mediators[19, 20], and hypovolemia from decreased fluid intake and increased insensible loss due to fever[21]. Muscle pain and tenderness mainly involve the calf and lumbar areas and there has been some evidence that muscle injury might be caused directly by leptospires during the early phase of infection [22] which could explain why calf pain might occur even before fever or other systemic symptoms. Jaundice, one of the most clinically recognizable features of leptospirosis, also known as leptospirosis icterohemorrhagica or Weil’s disease, results from cholestasis rather than hepatocellular damage as implied by a characteristic disproportion between the slight elevations in transaminase despite markedly high bilirubin levels.

Renal involvement is also notable in leptospirosis. According to Sitprija et al., the pathogenesis of renal complications is the result of direct invasion of leptospires, hemodynamic changes, and inflammation [23]. In severe cases of leptospirosis, non-specific inflammatory factors including hemolysis, myonecrosis, intravascular coagulation, free radicals, hyperbilirubinemia and increased blood viscosity additionally contribute to impaired renal function [19]. The renal pathology of AKI from leptospirosis has more extensive damage than other diseases, especially at proximal convoluted tubules [24].

Electrolyte disturbances are common features of tropical diseases. Hyponatremia in leptospirosis is attributed to several causes, including urinary sodium loss, cellular influx of sodium due to decreased Na^+^/ K^+^-APTase activity, increased level of ADH, and resetting of osmoreceptors [25]. Hypokalemia and polyuria are notable in leptospirosis and can be explained by a decrease in the expression of sodium/hydrogen exchanger isoform 3 (NHE 3), aquaporin 1, and α-Na^+^/K^+^-ATPase in proximal convoluted tubule cells [24]. On the other hand, aquaporin 2 expression in the collecting tubules is enhanced in leptospirosis kidneys compared to non-leptospirosis kidneys [24].Therefore, although hyponatremia and hypokalemia can separately occur in many tropical diseases [25, 26] hyponatremia together with hypokalemia was characteristically found in leptospirosis (Fig 2).

Pulmonary involvement has been reported in 20–70% of leptospirosis patients. Chest radiographs of leptospirosis are characterized primarily by diffuse small opacities that are disseminated or coalesced into larger areas of consolidation [27]. Despite the presence of clinical symptoms, pulmonary hemorrhage may also be concealed with few or no chest radiographic abnormalities [28]. Andrade et al. postulated that leptospirosis infection could cause a decrease in epithelial sodium channel (ENaC) and an increase in Na-K-Cl cotransporter (NKCCl) expression that dissipate the osmotic gradient of Na between the alveolar lumen and the interstitium. This leads to increased pulmonary permeability such as that observed in acute respiratory distress syndrome (ARDS) [29].

Low hemoglobin, a common finding in leptospirosis that has not been emphasized in literature, is caused by several factors including blood loss and hemolysis [30]. Although the exact mechanism of abnormal bleeding is not currently well understood, several facilitating factors may be thrombocytopenia, capillary endothelial damage, and coagulation defects resulting from hepatic dysfunction, consumptive coagulopathy and disseminated intravascular coagulation [31].

This was a prospective, multicenter study that used 3 standard confirmatory tests for leptospirosis: MAT, direct culture, and PCR. Additionally, the study had both a development and a validation cohort. However, our study has some limitations. First, the patients enrolled in the study were from an endemic area of leptospirosis which may limit the generalizability of the THAI-LEPTO score in non-endemic areas. Secondly, the score should be applied only after excluding other known infectious diseases, as the study was only conducted in leptospirosis suspected cases without other infectious diseases. Thirdly, most of the enrolled cases had severe clinical manifestations upon admission limiting the applicability of the score to severe leptospirosis suspected cases.

To conclude, THAI-LEPTO score is a newly developed diagnostic tool for early presumptive diagnosis of leptospirosis in patients presenting with severe clinical suspicion of the disease. Each predictor used in the score has been supported by evidence of clinical and pathophysiological correlation. However, an additional large validation study may be needed to evaluate the usefulness in general practice and in patients from non-endemic areas.

## Supporting information

S1 Fig The proposed diagram of simplified THAI-LEPTO score (8 variables) in clinical practice at the point of care. ^a^Clinical criteria of leptospirosis according to WHO clinical description as the usual presentation is an acute febrile illness with headache, myalgia (particularly calf muscle) and prostration associated with any of the following symptoms/signs: conjunctival suffusion, anuria or oliguria, jaundice, cough; hemoptysis and breathlessness, hemorrhages (from the intestines; lung bleeding is notorious in some areas), meningeal irritation, Cardiac arrhythmia of failure, skin rash ^b^For cutoff value of 5; the Sensitivity, Specificity, Positive likelihood ratio and Post-Test Probability are 0.794, 0.711, 2.740, and 0.733, respectively. For in detail of other cutoff values of simplified and original THAI-LEPTO Score models see Table 4. ^c^Other specific diseases such as hepatobiliary tract infection, bacterial sepsis, and malaria must be ruled out before making a presumptive diagnosis of leptospirosis. ^e^Definition of each factor: “Clinical Jaundice”; yellowish pigmentation of the skin, the sclera, and other mucous membranes, “Clinical Hypotension”; mean arterial pressure lower than 70 mm Hg or symptomatic low blood pressure that needed volume resuscitation or vasopressor, “Hb < 12”; hemoglobin < 12 g/dL, “AKI”; according to KDIGO criteria for acute kidney injury as increase in serum creatinine by ≥0.3 mg/dL (≥26.5 μmol/L) within 48 hours; or increase in serum creatinine to ≥1.5 times baseline, which is known or presumed to have occurred within the prior seven days; or urine volume < 0.5 mL/kg/h for six hours, “Muscle pain”; non-traumatic-sore aching muscles that are commonly involve the calves and lower back, “K < 3.5 & Na < 135”; Potassium < 3.5 mEq/L combined with Sodium < 135 mEq/L, “PMNs ≥ 80 & WBC < 10,000 cells/μL”; polymorphonuclear leucocytes ≥ 80% combined with white blood cells count < 10,000 /μL in a complete blood counts (CBC) test, “Pulmonary opacity”; on a chest X-ray lung abnormalities with increased density in any pattern such as consolidation, interstitial, or nodular pattern, that they may consist of nonspecific, diffuse, small opacities, which may be disseminated or coalesce in to larger area of consolidation such as in pulmonary edema, ARDS or lung hemorrhage.
(TIF)
