# Supplementary figures and images for: Thai-Lepto-on-admission probability (THAI-LEPTO) score as an early tool for initial diagnosis of leptospirosis: Result from Thai-Lepto AKI study group
Source: PLoS Negl Trop Dis. 2018 Mar 19;12(3):e0006319. doi: 10.1371/journal.pntd.0006319 (PMC5875898; doi:10.1371/journal.pntd.0006319)

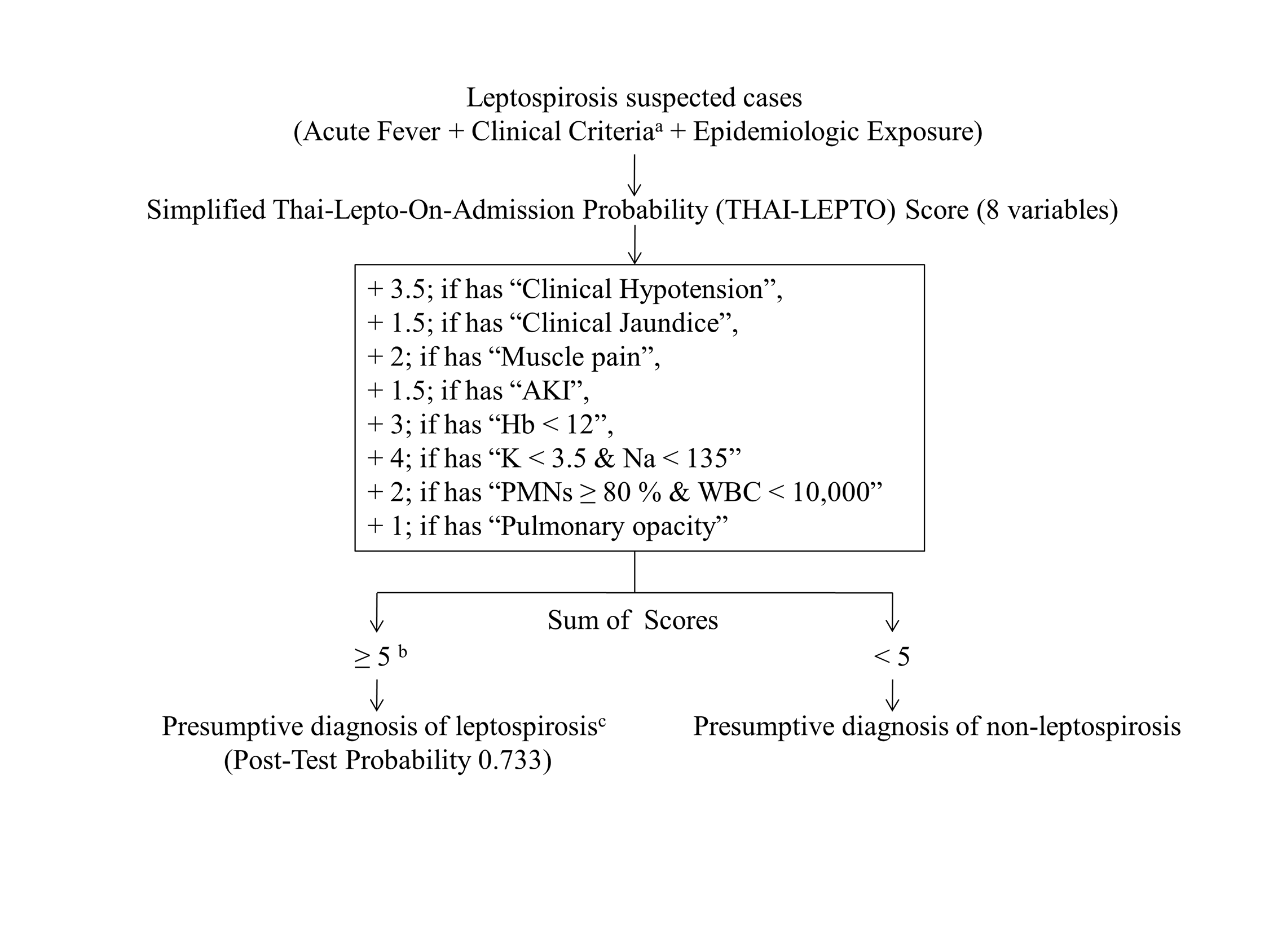

Supplement: S1 Fig — aClinical criteria of leptospirosis according to WHO clinical description as the usual presentation is an acute febrile illness with headache, myalgia (particularly calf muscle) and prostration associated with any of the following symptoms/signs: conjunctival suffusion, anuria or oliguria, jaundice, cough; hemoptysis and breathlessness, hemorrhages (from the intestines; lung bleeding is notorious in some areas), meningeal irritation, Cardiac arrhythmia of failure, skin rash bFor cutoff value of 5; the Sensitivity, Specificity, Positive likelihood ratio and Post-Test Probability are 0.794, 0.711, 2.740, and 0.733, respectively. For in detail of other cutoff values of simplified and original THAI-LEPTO Score models see Table 4. cOther specific diseases such as hepatobiliary tract infection, bacterial sepsis, and malaria must be ruled out before making a presumptive diagnosis of leptospirosis. eDefinition of each factor: “Clinical Jaundice”; yellowish pigmentation of the skin, the sclera, and other mucous membranes, “Clinical Hypotension”; mean arterial pressure lower than 70 mm Hg or symptomatic low blood pressure that needed volume resuscitation or vasopressor, “Hb < 12”; hemoglobin < 12 g/dL, “AKI”; according to KDIGO criteria for acute kidney injury as increase in serum creatinine by ≥0.3 mg/dL (≥26.5 μmol/L) within 48 hours; or increase in serum creatinine to ≥1.5 times baseline, which is known or presumed to have occurred within the prior seven days; or urine volume < 0.5 mL/kg/h for six hours, “Muscle pain”; non-traumatic-sore aching muscles that are commonly involve the calves and lower back, “K < 3.5 & Na < 135”; Potassium < 3.5 mEq/L combined with Sodium < 135 mEq/L, “PMNs ≥ 80 & WBC < 10,000 cells/μL”; polymorphonuclear leucocytes ≥ 80% combined with white blood cells count < 10,000 /μL in a complete blood counts (CBC) test, “Pulmonary opacity”; on a chest X-ray lung abnormalities with increased density in any pattern such as consolidation, interstitial, or [file pntd.0006319.s001.tif]
